# New Means of Relieving Tooth Ache

**Published:** 1873-12

**Authors:** 


*New Means of Relieving Toothache.*—In the *Revue Medicale de Toulouse*, we find the following:

This remedy consists in the employment of injections introduced into the gum near the diseased tooth. Dr. Dop has tried these injections in about one hundred cases. In twenty cases he made use of morphia, which succeeded very well, but only for a time. Chloroform was far more successful, and is now exclusively used by Dr. Dop. It was eminently successful in sixty-two cases out of eighty. The injection is made with the small syringe commonly used in France for subcutaneous injections. Only two drops are put in at a time. The needle is introduced gradually, and must remain *in situ* a few seconds. On withdrawing it, pressure must be exerted on the gum with the finger. In by far the greater number of cases, one injection is quite enough to stop the toothache. In some instances a second injection is needed twenty-four hours after the first. During absence of pain the tooth may be plugged or dressed with creosote.

